# Analysis of postpartum hypertension in women with preeclampsia

**DOI:** 10.1038/s41371-023-00849-3

**Published:** 2023-07-22

**Authors:** Qinqin Xue, Guang Li, Yanyun Gao, Yunjing Deng, Bianju Xu, Yu Chen, Yu Gao, Qi Chen

**Affiliations:** 1 Department of Obstetrics and Gynaecology, Yulin First Hospital, Yulin City, Shanxi Province China; 2 Department of Obstetrics and Gynaecology, The University of Auckland, Auckland, New Zealand

**Keywords:** Medical research, Disease prevention

## Abstract

Postpartum hypertension including persistent and recurrent hypertension could significantly affect maternal morbidity in preeclampsia. Data on the postpartum management of women with preeclampsia is limited. The objective of this study was to investigate the details of women experiencing persistent postpartum hypertension (PerPPH) or developing recurrent postpartum hypertension (RecPPH) after birth and whether the treatment with anti-hypertensive drugs could shorten the hospital stay. We also compared the clinical parameters in women who developed RecPPH and who did not. Data on 188 preeclamptic women, including the severity or time of onset, duration of hospital admission postpartum, and blood pressure during the admission were collected and analyzed. Overall, 30% of preeclamptic women developed RecPPH on day 1, 13% on day 3, and 12% on day 5 after birth. Women with severe preeclampsia or early onset preeclampsia are more likely to develop RecPPH, compared to women with mild or late onset preeclampsia. The overall time in days before discharge was not different between women with normal blood pressure and women with abnormal blood pressure 1 h after birth, regardless of the severity or gestation of onset. However, women with severe or early onset preeclampsia stayed longer in the hospital, compared to women with mild or late onset preeclampsia. In addition, women with severe or early onset preeclampsia or early delivery increased risk of developing RecPPH. In conclusion, we demonstrate that RecPPH became apparent on day 1 after delivery, and hence close monitoring of blood pressure even if initially seemingly normal after birth is important.

## Introduction

Preeclampsia, a pregnancy-specific disorder of hypertension affects 3–8% of all pregnancies worldwide and is a leading cause of maternal and neonatal mortality and morbidity [1]. Growing evidence suggests that there is an increased risk of developing recurrent preeclampsia and cardiovascular disease later in a woman’s life if she has had preeclampsia [2–4]. To date, there is no effective treatment for preeclampsia, except for the delivery of the placenta and fetus. Although blood pressure returns to normal range within a few days after delivery in most women with preeclampsia, a significant proportion of preeclamptic women experience postpartum hypertension or remain persistent postpartum hypertension (PerPPH), especially within the first 10 days after birth [5]. This includes some preeclamptic women who develop recurrent postpartum hypertension (RecPPH) following normal blood pressure recording at the time of delivery [6–8].

The complications of postpartum hypertension have been well-recognized and specific recommendations to closely monitor and/or control of blood pressure after delivery have been suggested [9]. These studies strongly suggested that interventions for women with postpartum hypertension are urgently needed. A recent randomized control trial reported that treatment with furosemide in a short course would improve postpartum blood pressure control [10], suggesting that is a potentially important treatment option [11].

There is currently a paucity of studies focusing on postpartum management in women with preeclampsia, including how to manage the blood pressure after birth, due in part to there not being many studies reporting the trends in blood pressure postpartum in women with preeclampsia. Although a study recommended a necessary treatment if preeclamptic women had systolic blood pressure ≥150 mmHg or diastolic blood pressure ≥90 mmHg after delivery [12], our hospital guideline suggests a continuous treatment with anti-hypertensive drugs when the blood pressure does not return to normal range (systolic blood pressure <140 mmHg or diastolic blood pressure <90 mmHg) after birth in women with preeclampsia. In addition, our hospital guideline recommends close monitoring of blood pressure in preeclamptic women after birth, with measurements taken every 4 to 6 h a day.

Continuous treatment for women with preeclampsia may decrease maternal morbidity and re-admission to the hospital and may reduce duration of postpartum admission after delivery. Longer hospital stays increase the cost of medical care in addition to the impact of hospitalization on the puerperium. Therefore, in this retrospective observational study with a relatively large sample size, we aimed to investigate (1) the details of preeclamptic women experiencing PerPPH or developing RecPPH after delivery; (2) the effects of anti-hypertensive treatment on the length of hospital stay in preeclamptic women with abnormal blood pressure after birth, taking account into the severity and gestation of onset of preeclampsia; (3) the differences in clinical parameters between preeclamptic women who experienced PerPPH or developed RecPPH and who did not after birth.

## Material and methods

This study has been approved by the Ethics Committee of the Yulin First Hospital, China (reference number: 2022-005). The Ethics Committee of the Yulin First Hospital, China has waived the need for individual patient informed consent for this study due to its retrospective nature. This investigation conforms to the principles outlined in the declaration of Helsinki.

### Study cohort and clinical data collection

Women who were diagnosed with preeclampsia, without a history of chronic hypertension or renal or cardiovascular disease from January 2019 to December 2021 at Yulin First hospital in Shanxi province, China were included. Data on maternal age at diagnosis of preeclampsia, parity, gravidity, maternal body mass index (BMI), systolic and diastolic blood pressure at diagnosis, at 1 h after birth, on the first or third or fifth postpartum days, and at discharge, gestational age at diagnosis, gestational age at delivery, days to discharge after birth and birthweight were collected from the hospital electronic database. The blood pressure was measured every 4 h a day when the patients stayed in the hospital. Blood pressure after birth was measured after 1 h of giving a birth.

Preeclampsia was defined as a maternal systolic blood pressure ≥140 mmHg and/or diastolic blood pressure ≥90 mmHg measured on two occasions separated by at least 6 h, with or without proteinuria (>300 mg in a 24 h period), and/or impaired liver function and/or lower platelet count, after 20 weeks of gestation by the guidelines of the International Society for the Study of Hypertension in Pregnancy (ISSHP) [13]. The gestation of onset before and after 34 weeks gestation was defined as early or late onset preeclampsia. Systolic blood pressure ≥160 mmHg and/or diastolic blood pressure ≥110 mmHg with impaired liver or renal function and/or lower platelet count was defined as severe preeclampsia [13].

The anti-hypertensive drugs, nifedipine (10 mg/8 h) or labetalol (30 mg/8 h) were given to all women when their blood pressure did not return to normal range (systolic blood pressure ≤140 mmHg, or diastolic blood pressure ≤90 mmHg) after birth. The anti-hypertensive drugs were also given to women when they developed RecPPH after birth.

RecPPH was defined as systolic blood pressure reaching ≥140 mmHg or diastolic blood pressure reaching ≥90 mmHg again after giving birth, when their blood pressure was returned to a normal range 1 h after delivery. The criteria for discharge in our hospital guidelines include that blood pressure in preeclamptic women remains stable or improved to normal range (systolic blood pressure ≤140 or diastolic blood pressure ≤90 mmHg) and biomarkers of liver and kidney function returned to normal range. Postpartum hypertension was defined as pregnant women having abnormal blood pressure (systolic blood pressure ≥140 or diastolic blood pressure ≥90 mmHg) at least once, after birth until discharge.

### Statistical analysis

**Fig. 1:**
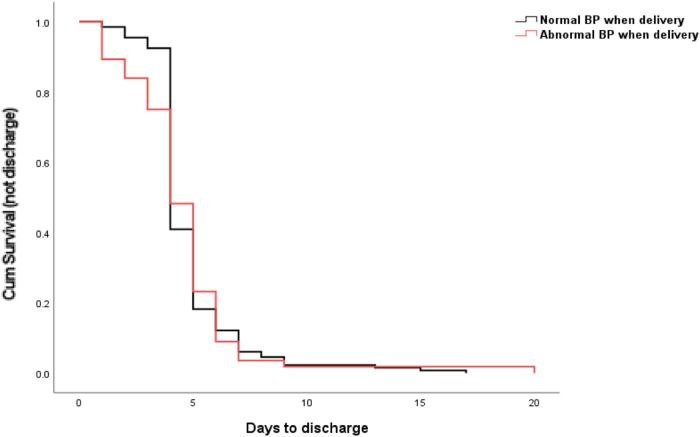
The comparison of days to discharge between the groups. Kaplan–Meier curve showing the days to discharge was no difference in preeclamptic women with normal and abnormal blood pressure at the time of birth.

**Fig. 2:**
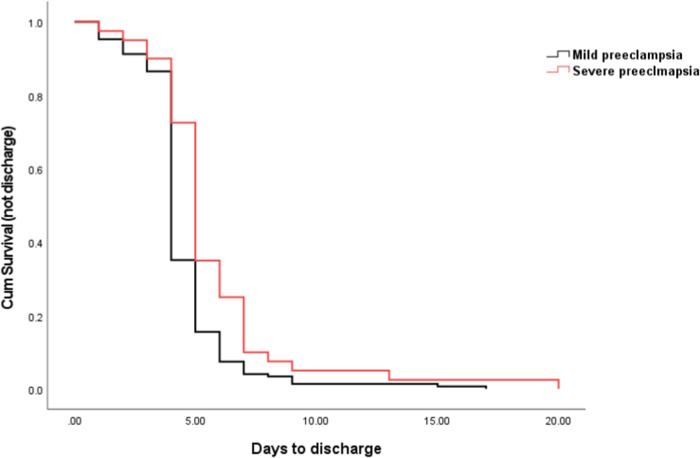
The comparison of days to discharge between severe and mild preeclampsia. Kaplan–Meier curve showing the days to discharge was significant longer in severe preeclampsia, compared to mild preeclampsia.

**Fig. 3:**
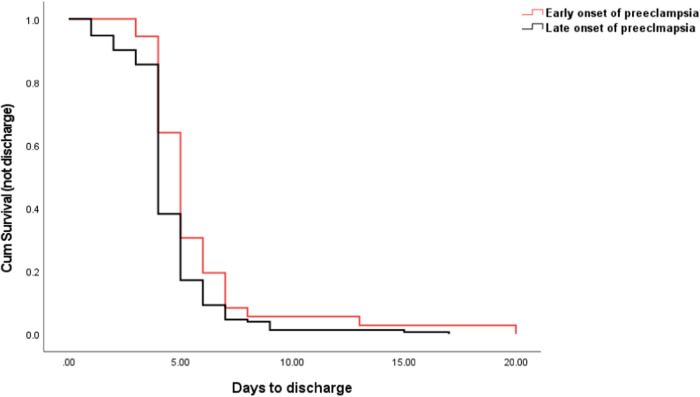
The comparison of days to discharge between early onset and late onset preeclampsia. Kaplan–Meier curve showing the days to discharge was significant longer in early onset preeclampsia, compared to late onset preeclampsia.

Data on tables are presented as mean and standard deviation (SD), number (percentage) when it is appropriate. Nonparametric test (Mann–Whitney test), Chi-square test or odds ratio (OR) were performed for the assessment of statistical differences between the groups, as appropriate, using GraphPad Prism software (version 9.2). OpenEpi (version 3.1) was used for calculation of percentage and 95% Confidence Intervals (CI). The Kaplan–Meier curve for comparing the days to discharge was performed using SASS (version 9.4) for Figs. 1–3. A *p* < 0.05 was considered statistically significant.

## Results

In this study, a total of 188 women with preeclampsia were included. The clinical characteristics of the study cohort are summarized in Table 1. The mean maternal age was 30.1 ± 3.85 years. Forty women were diagnosed with severe preeclampsia, and 36 women were diagnosed with early onset preeclampsia. The mean gestational age at delivery was 37^+5^ ± 2.6 and the mean birthweight was 2976 ± 766 g. The mean number of days to discharge after birth was 4.7 days.

**Table 1:** Clinical parameters in the study cohort (*n* = 188).

Maternal age at diagnosis (years, mean/SD)	30.1 ± 3.85
Parity (*n*, %)	
1 2 ≥3	107 (57%) 63 (33%) 18 (10%)
Gravidity (*n*, %)	
1 2 ≥3	75 (40%) 53 (28%) 62 (32%)
BMI (kg/m^2^, mean/SD)	27.7 ± 4.9
Systolic (mmHg, mean/SD)	149 ± 12.6
Diastolic (mmHg, mean/SD)	97 ± 9.8
Birthweight (g, mean/SD)	2976 ± 766
Gestational age at delivery (weeks, mean/SD)	37^+5^ ± 2.6
Gestational age at onset (weeks, mean/SD)	36^+1^ ± 3.4
Days to discharge after delivery (day, mean/SD)	4.74 ± 2.3
Mode of delivery	
Vaginal delivery (*n*, %) Caesarean section (*n*, %)	80 (42%) 108 (58%)

Overall, there were 57 (29%) preeclamptic women whose blood pressure 1 h after birth did not return to the normal range (Table 2). There were significantly more women with severe preeclampsia having abnormal blood pressure measured 1 h after birth, compared to women with mild preeclampsia (45% vs 26%, *p* < 0.05, Chi-square test). There were also significantly more women with early onset preeclampsia having abnormal blood pressure 1 h after birth, compared to women with late onset preeclampsia (44% vs 27%, *p* < 0.05, Chi-square test).

**Table 2:** The trend of abnormal blood pressure in preeclamptic women after birth (*n* = 188), expressed as percentage and 95% confidence intervals (CI).

Overall			
One hour after birth (*n* = 188)	Day 1 after birth (*n* = 188)	Day 3 after birth (*n* = 173)^a^	Day 5 after birth (*n* = 86)^b^
*N* = 57 (29%) (24.84, 37.43)	*N* = 89 (47%) (40.03, 57.74) PerPPH *n* = 34 (38%) (28.1, 49.1) RecPPH *n* = 55 (62%) (50.8, 71.9)	*N* = 105 (61%) (52.92, 68.03) PerPPH *n* = 83 (79%) (70.01, 86.5) RecPPH *n* = 22 (21%) (13.6, 29.9)	*N* = 52 (60%) (49.34, 70.85) PerPPH *n* = 46 (88%) (76.5, 95.6) RecPPH *n* = 6 (12%) (4.3, 2.34)
**Severe preeclampsia**			
**One hour after birth (*n = 40)***	**Day 1 after birth (*n* = 40)**	**Day 3 after birth (*n* = 38)**	**Day 5 after birth (*n* = 30)**
*N* = 18 (45%) (29.26, 64.51)	*N* = 26 (65%) (48.3, 79.4) PerPPH *n* = 11 (42%) (23.3, 63.1) RecPPH *n* = 15 (58%) (36.9, 76.6)	*N* = 29 (76%) (59.7, 88.6) PerPPH *n* = 26 (89%) (72.6, 97.8) RecPPH *n* = 3 (11%) (2.2, 27.3)	*N* = 16 (53%) (34.3, 71.6) PerPPH *n* = 15 (94%) (69.7, 99.8) RecPPH *n* = 1 (6%) (0.15,30.2)
**Mild preeclampsia**			
**One hour after birth (*n* = 148)**	**Day 1 after birth (*n* = 148)**	**Day 3 after birth (*n* = 135)**	**Day 5 after birth (*n* = 56)**
*N* = 39 (26%) (19.46, 34.22)*	*N* = 63 (42%) (34.5, 50.9) PerPPH *n* = 23 (36%) (24.7, 49.6) RecPPH *n* = 40 (64%) (50.4, 75.3)	*N* = 76 (52%) (47.5, 64.8) PerPPH *n* = 56 (74%) (62.3, 83.1) RecPPH *n* = 20 (26%) (16.8, 37.7)	*N* = 36 (64%) (60.4, 76.6) PerPPH *n* = 31 (86%) (70.5, 95.3) RecPPH *n* = 5 (14%) (4.7, 29.5)
*p **< 0.05, compared to severe preeclampsia (Chi-square test)**			
**Early onset preeclampsia**			
**One hour after birth (*n* = 36)**	**Day 1 after birth (*n* = 36)**	**Day 3 after birth (*n* = 36)**	**Day 5 after birth (*n* = 22)**
*N* = 16 (44%) (27.9, 61.9)	*N* = 26 (72%) (54.8, 85.8) PerPPH *n* = 13 (50%) (29.9, 70.1) RecPPH *n* = 13 (50%) (29.9, 70.1)	*N* = 27 (75%) (57.8, 87.8) PerPPH *n* = 25 (93%) (75.7, 99.1) RecPPH *n* = 2 (7%) (0.91, 24.3)	*N* = 15 (68%) (45.1, 86.1) PerPPH *n* = 14 (93%) (68.1, 99.8) RecPPH *n* = 1 (7%) (0.16, 31.9)
**Late onset preeclampsia**			
**One hour after birth (*n* = 152)**	**Day 1 after birth (*n* = 152)**	**Day 3 after birth (*n* = 137)**	**Day 5 after birth (*n* = 64)**
*N* = 41 (27%) (20.1, 34.7)*	*N* = 64 (42%) (34.1, 50.4) PerPPH *n* = 21 (33%) (21.6, 45.6) RecPPH *n* = 43 (67%) (54.3, 78.4)	*N* = 80 (58%) (49.6, 66.7) PerPPH *n* = 55 (69%) (57.4, 78.6) RecPPH *n* = 25 (31%) (21.3, 42.6)	*N* = 37 (57%) (44.8, 70.1) PerPPH *n* = 32 (86%) (71.2, 95.5) RecPPH *n* = 5 (14%) (4.53, 28.77)

PerPPH reflected the maintenance of hypertension detected in the previous time point. RecPPH reflected a high blood pressure value reaching in spite that levels had returned to normal either 1 h after birth or thereafter.

*p < 0.05, compared to early onset preeclampsia (Chi-square test)

^a^15 cases were discharged on day 2 after birth.

^b^additional 87 cases were discharged on day 4 after birth.

On day 1 after birth, 89 (47%) women had abnormal blood pressure. Of these, 55 (62%) women developed RecPPH (Table 2). On day 3 after birth, 105 (61%) women had abnormal blood pressure, and 15 women were discharged on day 2 after delivery. Of those 105 cases, 22 (21%) women developed RecPPH (Table 2). On day 5 after birth, there were only 86 preeclamptic women staying in the hospital, and of them, 52 (60%) women had abnormal blood pressure and 6 (12%) of these women developed RecPPH (Table 2). As shown in Table 2, more preeclamptic women developed RecPPH on day 1 after birth, even though they had normal blood pressure 1 h after birth regardless of the severity, or gestation of onset of preeclampsia.

Overall, there was no difference in the days to discharge after birth between preeclamptic women with normal blood pressure and preeclamptic women with abnormal blood pressure 1 h after birth (4.82 vs 4.55 days, *p* = 0.733, Fig. 1).

Preeclampsia divides into severe and mild forms. We then compared the days to discharge according to the severity of preeclampsia. As shown in Fig. 2, the mean number of days to discharge in women with severe preeclampsia was 5.7 days, which was significantly longer than the mean number of days to discharge in women with mild preeclampsia (4.5 days, *p* < 0.001). We also then compared the days to discharge according to the gestation of onset. As shown in Fig. 3, the mean number of days to discharge in women with early onset preeclampsia was 5.7 days, which was significantly longer than the mean number of days to discharge in women with late onset preeclampsia (4.5 days, *p* < 0.001).

We then analyzed the days to discharge between preeclamptic women with normal blood pressure and with abnormal blood pressure 1 h after birth, according to the severity, or gestation of onset of preeclampsia (Table 3). There were 18 women with severe preeclampsia whose blood pressure did not return to normal range measured 1 h after birth. The mean number of days to discharge after birth of these women was 5.45 ± 2.32 days, which was no different from severe preeclamptic women with normal blood pressure after birth (6.0 ± 3.80 days, *p* = 0.904). There were 38 women with mild preeclampsia whose blood pressure 1 h after birth did not return to the normal range. The mean number of days to discharge after birth of these women was 3.86 ± 1.69 days, which was no different from mild preeclamptic women with a normal blood pressure after birth (4.69 ± 2.0 days, *p* = 0.177). There were 16 women with early onset preeclampsia whose blood pressure did not return to normal range after birth. The mean number of days to discharge after birth of these women was 6.0 ± 3.89 days, which was no different from severe preeclamptic women with a normal blood pressure at the time of delivery (5.35 ± 2.18 days, *p* = 0.604). There were 41 women with late onset preeclampsia whose blood pressure did not return to normal range after birth. The mean number of days to discharge after birth in those women was 4.0 ± 1.83 days, which was no different from mild preeclamptic women with normal blood pressure after birth (4.72 ± 2.05 days, *p* = 0.191).

**Table 3:** Days to discharge between preeclamptic women with normal blood pressure and with abnormal blood pressure measured 1 h after birth, according to the severity, or the time of onset of preeclampsia.

Sub-groups		Days to discharge (days, mean/SD)	*p-*value (Mann–Whitney test)
Severe preeclampsia (*n* = 40)	Normal blood pressure (*n* = 22)	5.45 ± 2.32	0.904
	Abnormal blood pressure (*n* = 18)	6.0 ± 3.80	
Mild preeclampsia (*n* = 148)	Normal blood pressure (*n* = 107)	4.69 ± 2.00	0.177
	Abnormal blood pressure (*n* = 41)	3.86 ± 1.69	
Early onset preeclampsia (*n* = 36)	Normal blood pressure (*n* = 20)	5.35 ± 2.18	0.604
	Abnormal blood pressure (*n* = 16)	6.0 ± 3.89	
Late onset preeclampsia (*n* = 152)	Normal blood pressure (*n* = 111)	4.72 ± 2.05	0.191
	Abnormal blood pressure (*n* = 41)	4.0 ± 1.83	

We also compared the days to discharge between severe and mild preeclampsia or between early onset and late onset preeclampsia. As shown in Table 2, 75% (30 out of 40 cases) of severe preeclampsia stayed in the hospital for more than 5 days, which was significantly higher than mild preeclampsia (56 out of 148 cases, 38%, *p* < 0.001, Chi-square test). Also shown in Table 2, 61% (22 out of 36 cases) of early onset preeclampsia stayed in the hospital for more than 5 days, which was significantly longer than those with late onset preeclampsia (63 out of 152 cases, 41%, *p* < 0.001, Chi-square test).

We next compared the clinical parameters between preeclamptic women with normal blood pressure 1 h after birth and those with an abnormal blood pressure 1 h after birth, to identify potential factors contributing to the observed changes in blood pressure after delivery (Table 4). Women with severe preeclampsia were more likely to develop RecPPH (OR 4.111, 95% CI: 1.236 to 13.271, *p* = 0.016)), compared to women with mild preeclampsia. In addition, women with early onset preeclampsia were more likely to developed RecPPH (OR 3.539, 95% CI: 1.049 to 11.472, *p* = 0.034)), compared to women with late onset preeclampsia. The mean gestation at delivery in preeclamptic women without postpartum hypertension was 38^+5^ ± 1.5, which was significantly longer than that of preeclamptic women with postpartum hypertension (37^+3^ ± 2.8, *p* = 0.002). BMI did not affect the risk of developing postpartum hypertension (*p* = 0.972). Preeclamptic women with more than 2 live births were found to have a higher risk of RecPPH than those who had had one previous live birth (OR 2.766, 95% CI: 1.257 to 5.837, *p* = 0.009).

**Table 4:** The comparison of clinical parameters in women who developed recurrent postpartum hypertension and women who did not.

	With postpartum hypertension (*n* = 148)	Without postpartum hypertension (*n* = 40)	*p-*value (Mann–Whitney test)
Maternal age at diagnosis (years, mean/SD)	30.2 ± 4.0	29.3 ± 3.0	*P* = 0.100
BMI (kg/m^2^, mean/SD)	27.7 ± 4.9	27.59 ± 4.9	*P* = 0.972
Delivery weeks (mean/SD)	37^+3^ ± 2.8	38^+5^ ± 1.5	*P* = 0.002
			OR (95% CI)
Severe preeclampsia (*n*, row %) Mild preeclampsia (*n*, row %)	37 (92%) 111 (75%)	3 (8%) 37 (25%)	4.111 (1.236, 13.27) *P* = 0.016
Early onset preeclampsia (*n*, row %) Late onset preeclampsia (*n*, row %)	33 (92%) 115 (76%)	3 (8%) 37 (24%)	3.539 (1.049, 11.47) *P* = 0.04
Parity			
*P* = 1 (*n*, row %) *P* ≥ 2 (*n*, row %)	77 (72%) 71 (88%)	30 (28%) 10 (12%)	2.766 (1.257, 5.837) *P* = 0.009
Mode of delivery			
Vaginal delivery (*n*, row %) Caesarean section (*n*, row %)	59 (74%) 89 (82%)	21 (26%) 19 (18%)	0.599 (0.297, 1.223) *P* = 0.151

The definition of recurrent postpartum hypertension was that women developed abnormal blood pressure at least at one time point until the time of discharge.

## Discussion

In this retrospective observational study, we found that overall, 30% of the preeclamptic women developed RecPPH on the first postpartum day, 13% on the third and 12% on the fifth day. Women with severe preeclampsia or early onset preeclampsia or early delivery are more likely to develop RecPPH, compared to women with mild or late onset preeclampsia. Overall, the number of days to discharge were not different between preeclamptic women with normal blood pressure and those with an abnormal blood pressure measured 1 h after birth, regardless of the severity or gestation of onset. However, women with severe or early onset preeclampsia stayed longer in the hospital, compared to women with mild or late onset preeclampsia.

The blood pressure normally would reduce to a normal range after birth in women with preeclampsia, however, many preeclamptic women are found to continuously have an abnormal blood pressure or others develop RecPPH after having had a normal blood pressure immediately postpartum [5]. Restoration of vascular tone to pre-pregnancy levels may contribute to the blood pressure increase. Studies have reported that 30% of the cases of preeclampsia could develop postpartum hypertension, including PerPPH and RecPPH [14]. However, the details of this observation have not been fully investigated. In our current study, when we assessed the incidence of the development of RecPPH, we found that 30% of our cases of preeclampsia developed RecPPH on day 1 after birth. The percentage of RecPPH in women with abnormal blood pressure on day 1 after birth was 62%. On the other hand, 13% of women with preeclampsia developed RecPPH after day 3 of birth, showing that RecPPH most likely occurs in the first 24 h after birth. This observation was regardless of the severity, or gestation of onset of preeclampsia. Therefore, women with preeclampsia should be closely monitored till at least five days after birth, and our findings are consistent with other study [9].

The optimal time for the discharge of preeclamptic women after birth has not been well investigated, hence close monitoring is recommended for at least three days after birth [7–15]. A recent study reported that women with preeclampsia should stay in the hospital for 4 to 6 days [16]. In our current study, we found that the mean number of days to discharge was 4.74 days. In addition, the mean number of days to discharge was not associated with the blood pressure measured 1 h after birth. It was also found that women with severe, or early onset preeclampsia stayed longer in the hospital than those with mild or late onset preeclampsia, even though all received treatment with anti-hypertensive drugs. This is likely to be because of the slower reduction of blood pressure in the severe cases. Our finding was also consistent with a previous study [16].

The comparison between women who did or did not develop RecPPH does not appear to have been reported. In our current study, we found that women with severe or early onset preeclampsia would have abnormal blood pressure after birth. Women with severe preeclampsia or with early onset preeclampsia had an increased 4.1-fold or 3.5-fold risk, respectively, of experiencing a persistently abnormal blood pressure. In addition, in women who did not develop RecPPH, the gestational age was 1 week longer, compared to those who developed RecPPH. In contrast, in this study, BMI did not influence the experiences of PerPPH or RecPPH in women with preeclampsia.

There are some limitations in this study. The blood pressure measurements in women with preeclampsia were only monitored for a week, follow-up data, including a 6 week postpartum blood pressure recording were not available and future longer monitoring is required.

In conclusion, in this observational study, we demonstrated that 30% of preeclamptic women developed RecPPH on day 1 after birth and this was associated with the severity and gestation of onset of preeclampsia. The severity and gestation of onset of preeclampsia and the delivery weeks were associated with the risk of developing RecPPH. The days to discharge were not different between preeclamptic women with or without an abnormal blood pressure at the time of birth. However, women with severe preeclampsia, or with early onset preeclampsia stayed longer in the hospital, compared to mild or late onset preeclampsia. Closely monitoring blood pressure after birth to women with preeclampsia is important because a normal blood pressure at 1 h after birth did not predict need for subsequent anti-hypertensive treatment.

## Summary

### What is known about this topic

- There is a risk of developing RecPPH in women with preeclampsia.
- Giving anti-hypertensive treatment in preeclamptic women with RecPPH is necessary.

### What this study adds

- 30% of preeclamptic women experienced RecPPH on day 1 after birth.
- The incidence of RecPPH is associated with the severity or gestation of onset of preeclampsia.
- Treatment with anti-hypertensive drugs can reduce the days of stay in the hospital.

## Data Availability

The datasets used and/or analysed during the current study are available from the corresponding author upon reasonable request.
